# Post-donation satisfaction in kidney transplantation: a survey of living donors in Japan

**DOI:** 10.1186/s12913-019-4556-5

**Published:** 2019-10-26

**Authors:** Sayaka Kobayashi, Rie Akaho, Kazuya Omoto, Hiroki Shirakawa, Tomokazu Shimizu, Hideki Ishida, Kazunari Tanabe, Katsuji Nishimura

**Affiliations:** 1 Department of Psychiatry, Tokyo Women’s Medical University, School of Medicine, Tokyo, Japan; 2 Department of Psychiatry, Saitama Medical Center, Saitama Medical University, Kawagoe, Japan; 3 Department of Urology, Tokyo Women’s Medical University, School of Medicine, Tokyo, Japan; 4 Department of Urology, Toda Chuo General Hospital, Saitama, Japan; 5 Department of Urology, Tokyo Metropolitan Health and Medical Treatment Corporation Okubo Hospital, Tokyo, Japan; 6 Department of Organ Transplant Medicine, Tokyo Women’s Medical University, School of Medicine, Tokyo, Japan

**Keywords:** Post-donation satisfaction, Living kidney donors

## Abstract

**Background:**

No studies using a valid, standardized method to measure post-donation satisfaction levels among living kidney donors (LKDs) have been published.

**Methods:**

Donor satisfaction levels were measured using the Japanese version of the Client Satisfaction Questionnaire-8 (CSQ-8), a validated, self-report questionnaire. To identify factors related to post-donation satisfaction levels, we compared donors’ sociodemographic and psychological characteristics and health-related quality of life (HRQoL), using the Short Form-36 Health Survey (SF-36), as well as recipients’ clinical characteristics and SF-36 scores between donors with and without low satisfaction. In addition, donors’ perceptions of the donation results and transplant procedure were assessed using measures that we developed.

**Results:**

The mean (standard deviation [SD]) CSQ-8 score for the 195 participants was 26.9 (3.4). Twenty-nine (14.9%) respondents with total scores < 1 SD below the mean CSQ-8 score were placed into the low satisfaction group. Multiple logistic regression analysis demonstrated that lower perceptions of receiving adequate information prior to transplantation (odds ratio [OR] = 0.17; 95% confidence interval [CI] = 0.079–0.379; *p* < 0.001), lower optimism according to the Life Orientation Test (OR = 1.24; 95% CI = 1.045–1.470; *p* = 0.014), and increased serum creatinine levels in the paired recipient (OR = 0.05; 95% CI = 0.250–1.011; *p* = 0.054) independently increased the odds of having less satisfaction with donation.

**Conclusions:**

Our findings suggest that careful pre-donation education and more detailed informed consent may be needed, especially in LKDs with low constitutional optimism.

## Background

Healthy living kidney donors (LKDs) do not reap medical benefits from donation but stand to gain a sense of satisfaction through the contribution that they make to recovery of the recipient’s health. Therefore, post-donation satisfaction levels could be considered an important outcome for the donor.

Many health-related quality of life (HRQoL) studies have been conducted on post-donation outcomes for the donor. According to these studies, 93–97% of donors have said, “I would donate again, given another chance” [1–7]. These findings also mean that 3–7% of donors do not share this opinion, thus suggesting that some donors’ satisfaction levels are low. For example, in a large cross-sectional study of 1414 LKDs in Norway, 80.7% of donors answered “definitely”, 13.9% “probably”, 2.3% “don’t know”, 1.8% “probably not”, and 1.3% answered “definitely not” when asked whether they would donate again [6]. This question has primarily been used as a measure of post-donation satisfaction among LKDs in several studies. However, this question may have less to do with the decision-making process than it does with donors’ satisfaction with the recipient’s outcome [8]. A recent study using exploratory factor analyses demonstrated that donors’ satisfaction was composed of three factors (unmet donor expectations about donation, interference of donation with daily activities, and pain and discomfort), which were not differentiated in the abovementioned single question [9].

To our knowledge, there are no published studies that have used a valid, standardized method to measure post-donation satisfaction levels. Here, we used the Japanese version of the Client Satisfaction Questionnaire-8 (CSQ-8) [10, 11], a standardized measure for global client/patient satisfaction with health services and clinical care.

A systemic review of the psychosocial health of LKDs demonstrated that a small proportion of LKDs had adverse psychosocial outcomes, such as decreased psychological well-being (e.g., depression) and a decrease in HRQoL [12], which may lead to post-donation dissatisfaction. Optimism may positively affect psychological [13] and physical [14] aspects among LKDs. Furthermore, the decision-making process surrounding whether to donate may be crucial for post-donation psychosocial outcomes in LKDs [15]. In Japan, living organ donors are, in principle, limited to family members (blood relatives within six degrees of kinship or relatives by marriage within three degrees of kinship), which may affect decision-making based on Asian attitudes regarding family relationships [16, 17]. Lower-quality relationships between recipients and family members or feelings of unattractiveness that occur after donation may also result in donor dissatisfaction [12].

In this study, we followed a new approach to evaluate post-donation satisfaction using the CSQ-8, a standardized measure. We aimed to clarify the factors associated with post-donation satisfaction among LKDs, primarily family members.

## Methods

### Participant recruitment

**Fig. 1:**
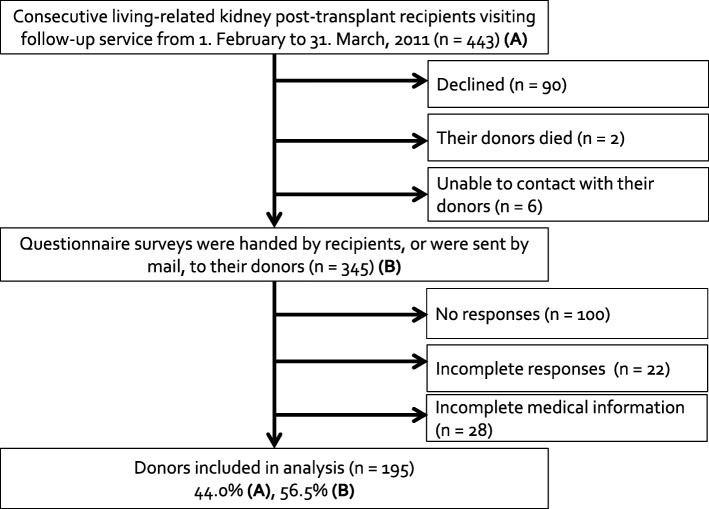
Flow diagram of participant recruitment

This study was conducted as part of a long-term HRQOL study of living, related kidney recipients and donors at our transplant center. We consecutively recruited 443 living, related kidney post-transplant recipients, who visited our follow-up clinic between 1 February and 31 March, 2011 to participate in our study. Of the 443 recipients, 90 declined and 353 agreed to participate. At the same time, we asked these recipients if we could request their paired donor to participate in our study. If they agreed, questionnaire surveys were administered to recipients or sent by mail to the paired donors. The paired donor of two recipients had died, and six recipients said that they could not contact their donors. Finally, questionnaire surveys were mailed or administered directly to a total 345 donors. Of these 345 donors, 100 did not respond, 22 provided incomplete surveys, and 28 surveys lacked medical information. Finally, 195 donors were included in the analysis (Fig. 1). This study was approved by the human ethics review board of Tokyo Women’s Medical University, and all participants signed a consent form.

### Assessment of post-donation satisfaction

All donors received a survey packet consisting of the following self-reporting tools. Donor satisfaction levels were measured using the Japanese version of the CSQ-8 [10, 11]. The CSQ-8 is a validated, self-report questionnaire for measuring satisfaction with a wide range of services and has been tested in numerous studies among diverse client/patient samples. The most extensive use of the CSQ-8 scale has been within mental health treatment, primary medical care, and a wide range of human service settings [18–20]. The CSQ-8 includes questions on the following eight topics (abbreviated), with response options provided on a 4-point Likert scale: quality of service received, received the desired service, respondents’ needs were met, would recommend to a friend, satisfied with the amount of help, deal more effectively with problems, satisfied with service, and would come back for service. The total possible score ranges from 8 to 32. Higher scores indicate greater satisfaction. To fit the context of LKDs, we added a note that “service” refers to “the whole process of living kidney transplantation, including your donation”.

To identify LKDs with low post-donation satisfaction, we classified the total CSQ-8 scores of participants into the following two groups: (a) low satisfaction group: < 1 standard deviation (SD) lower than the mean CSQ-8 score, and (b) non-low satisfaction group: ≥ 1 SD lower than the mean CSQ-8 score. Because the abovementioned response to the question “I would donate again, given another chance” could possibly underestimate post-donation dissatisfaction [9], we chose 1 SD below the mean CSQ-8 score as the cut-off point for low satisfaction.

### Assessment of health-related and psychosocial variables

The Short Form-36 Health Survey (SF-36) Japanese edition [21], a standardized self-reported questionnaire, was used to assess health-related quality of life (HRQoL). We used the Japanese version of the Zung Self-Rating Depression Scale (SDS) [22], a validated, self-reported, 20-questioninstrument, to assess psychological and somatic symptoms of depression. We used the Japanese version of the Life Orientation Test (LOT) [23], a valid, 12-item, 5-point scale instrument to assess individual differences in general optimism and pessimism.

Furthermore, using eight author-developed questions, we collected demographic information, including age at the time of survey, sex, time since donation, relationship to the recipient, total years of education, marital status, cohabitation status, and participants’ employment status.

### Donors’ perceptions of donation results and transplant procedure

We also assessed donors’ perception of the results of donation and transplantation procedure using a 13-item scale that we developed in other study (see Additional file 1) [24]. Items on this scale were extracted in a qualitative study of potential LKDs on the factors influencing decision-making when considering donation. Using data from 228 LKDs, these items were divided into 5 factors including 13 items, in factor analyses. These factors were: (1) good relationship with and support from family members; (2) adequate information prior to transplantation; (3) recipient’s recovery; (4) recipient’s gratitude toward the donor; (5) increase in self-esteem/self-worth after donation. The reliability of each factor has been confirmed, with good internal consistency.

### Assessment of paired recipients

Paired recipients also received a survey packet that included the CSQ-8, SF-36, SDS, and LOT. As an indicator of post-transplant physical condition, serum creatinine levels were collected from recipients at the time of the survey.

As mentioned, we classified the total CSQ-8 scores of participants into two groups according to satisfaction level, using a cut-off point 1 SD below the mean, which were used as dependent variables in the analysis. For the univariate analyses, a two-tailed test was used to identify differences between groups for continuous variables, and a chi-square test was used for categorical variables. To identify independent risk factors among donors with low satisfaction, multiple logistic regression analysis was performed, with forward stepwise variable selection. Variables from the univariate analyses with *p* < 0.1 were entered into a forward logistic regression model. Regression coefficients were used to calculate the odds ratio (OR) and 95% confidence interval (CI) of the OR. In all statistical analyses, *p* values < 0.05 were considered statistically significant. We performed all analyses using IBM SPSS Statistics, version 20 (IBM Corp., Armonk, NY, USA).

## Results

### Post-donation satisfaction levels

**Fig. 2:**
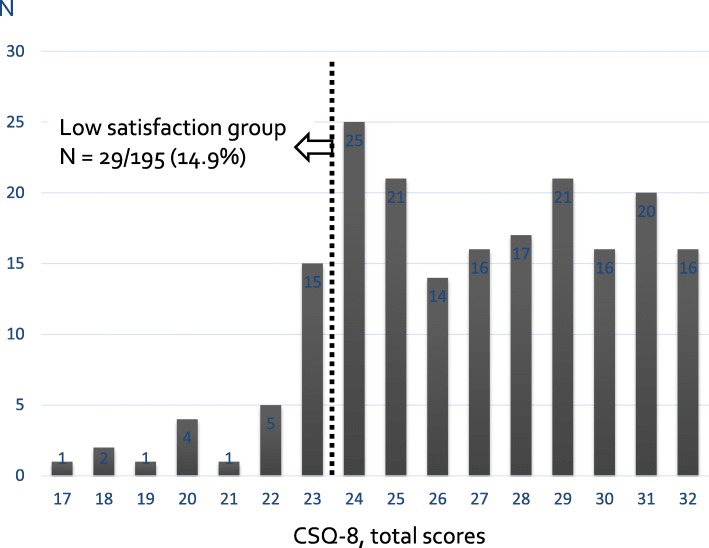
Distribution of Client Satisfaction Questionnaire-8 scores. Dashed line indicates 1 standard deviation below the mean score

**Fig. 3:**
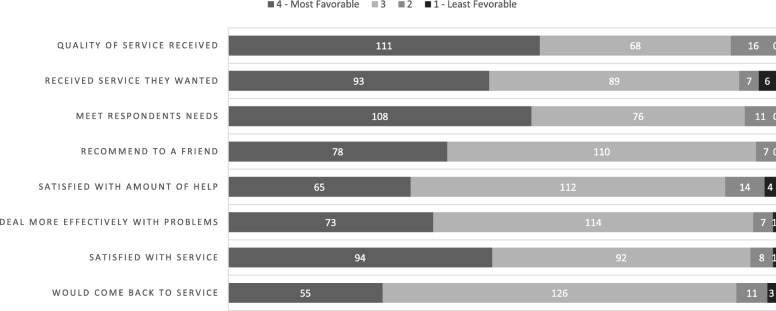
Distribution of each item (abbreviated) of the Client Satisfaction Questionnaire-8

The mean (SD) CSQ-8 score for the 195 participants was 26.9 (3.4). Twenty-nine (14.9%) participants were categorized into the low satisfaction group (Fig. 2). Distributions of each subscale score of the CSQ-8 are shown in Fig. 3. On the topic “would come back for service”, 14 respondents (7.2%) did not agree: 11 answered “No, I don’t think so” and 3 answered “No, definitely not”.

### Factors related to satisfaction levels

To identify factors related to post-donation satisfaction levels, we first used univariate analysis to compare donors’ sociodemographic, psychological, and health characteristics, and post-donation perceptions, as well as recipients’ clinical, psychological, and health characteristics between the groups with and without low levels of satisfaction (Table 1). Scores for post-donation perceptions of “receiving adequate information prior to transplant” (*p* = 0.001) and “increase in self-esteem/self-worth after donation” (*p* = 0.038) were significantly higher in the low satisfaction group than in the non-low satisfaction group (lower score indicates better perception). The Self-Rating Depression Scale score of donors was higher (*p* = 0.01), and the optimism score on the LOT of donors was significantly lower (*p* = 0.01) in the low satisfaction group than in the non-low satisfaction group.

**Table 1:** Relationship between satisfaction levels and sociodemographic, psychological, and health characteristics

	Donor satisfaction levels		*p*
	Low (*n* = 29)	Non-low (*n* = 166)	
*Donor variables*			
Age, years (mean ± SD)	61.5 ± 10.7	61.2 ± 9.6	0.887
Female sex	22 (75.9%)	113 (68.1%) 58.2 (1.0–833.3)	0.402
Time since donation, months	57.3 (3.8–205.3)		0.935
Relationship with recipient			0.949
Parent	15 (51.7%)	88 (53.0%)	
Child	0 (0%)	2 (1.2%)	
Sibling	3 (10.3%)	17 (10.2%)	
Spouse	9 (31.0%)	56 (33.7%)	
Education, ≤12 years	15 (51.7%)	88 (53.0%)	0.773
Married, current	21 (72.4%)	138 (83.1%)	0.254
Cohabitation, current			
With family	24 (82.8%)	147 (88.6%)	0.796
With recipient	17 (58.6%)	86 (51.8%)	0.340
With recipient prior to transplantation	18 (62.1%)	104 (62.7%)	0.899
Not employed, current	5 (17.2%)	28 (16.9%)	0.876
Perception of donation results and transplant procedure			
Good relationship with and support from family members	1.40 ± 0.49	1.28 ± 0.53	0.279
Adequate information prior to transplant	1.97 ± 0.78	1.41 ± 0.43	0.001^a^
Recipients’ recovery	1.41 ± 0.50	1.43 ± 0.61	0.908
Recipient’s gratefulness to the donor	1.41 ± 0.54	1.26 ± 0.47	0.109
Increase in self-esteem/self-worth through donation	1.59 ± 0.64	1.36 ± 0.54	0.038^a^
Self-Rating Depression Scale	39.1 ± 9.1	35.3 ± 6.7	0.01^a^
Life Orientation Test			
Optimism	13.7 ± 2.7	15.1 ± 2.6	0.01^a^
Pessimism	11.2 ± 3.1	11.2 ± 2.7	0.970
SF-36			
Physical function	87.8 ± 22.1	92.1 ± 12.1	0.122
Role — physical	83.8 ± 27.1	89.0 ± 20.7	0.24
Bodily pain	73.7 ± 34.1	77.5 ± 30.5	0.58
General health	73.5 ± 17.9	71.6 ± 18.6	0.605
Vitality	68.5 ± 18.0	71.8 ± 21.0	0.383
Social function	82.3 ± 24.4	84.9 ± 23.5	0.607
Role — emotional	89.4 ± 20.8	89.5 ± 20.1	0.973
Mental health	79.3 ± 13.7	79.4 ± 16.9	0.976
*Recipient variables*			
Age at transplantation, years	41.9 ± 13.6	41.8 ± 13.9	0.966
Dialysis duration before transplant, months	67.8 ± 68.9	53.5 ± 52.2	0.287
Serum creatinine level, current, mg/dL	1.5 ± 1.2	1.3 ± 0.4	0.095
CSQ-8	28.1 ± 3.0	29.2 ± 2.7	0.072
Self-Rating Depression Scale	36.3 ± 7.5	36.5 ± 7.3	0.873
Life Orientation Test			
Optimism	12.8 ± 2.8	13.4 ± 3.4	0.333
Pessimism	11.3 ± 2.7	11.4 ± 2.8	0.780
SF-36			
Physical function	92.1 ± 5.6	90.9 ± 9.1	0.476
Role — physical	67.2 ± 5.7	66.9 ± 15.0	0.896
Bodily pain	84.6 ± 12.5	81.5 ± 22.0	0.476
General health	58.1 ± 13.0	60.7 ± 16.5	0.424
Vitality	65.7 ± 10.8	67.6 ± 18.7	0.607
Social function	86.6 ± 13.4	84.8 ± 21.7	0.623
Role — emotional	65.8 ± 6.0	66.6 ± 16.7	0.814
Mental health	76.7 ± 4.9	77.2 ± 17.6	0.882

Data are presented as number (%) or median (SD), unless otherwise stated. Row percentages may not sum to 100 owing to rounding.

*CSQ-8* Client Satisfaction Questionnaire-8, *SF-36* MOS 36-item Short-Form Health Survey

^a^Significant variables

In the second step, we performed multiple logistic regression analysis using the forward stepwise selection method and six data sets, including serum creatinine level as well as CSQ-8 score of recipients, in addition to the above-mentioned variables that were significant in univariate analysis. Of the six variables, lower perceptions of “receiving adequate information prior to transplant” (OR = 0.17; 95% CI = 0.079–0.379; *p* < 0.001), lower optimism according to the LOT (OR = 1.24; 95% CI = 1.045–1.470; *p* = 0.014), and increased serum creatinine levels in the paired recipient (OR = 0.05; 95% CI = 0.250–1.011; *p* = 0.054) independently increased the odds of being in the low satisfaction group (Table 2).

**Table 2:** Multiple logistic regression analysis for predictors of donor satisfaction

	B	Wald	Exp (B)	95% CI for Exp (B)		*p*
				Lower	Upper	
*Donor variables*						
Perception of donation results and transplant procedure Adequate information prior to transplant*	−1.753	19.258	0.173	0.079	0.379	0.000
Life Orientation Test Optimism	0.215	6.084	1.240	1.045	1.470	0.014
*Recipient variables*						
Serum creatinine level, current	−.687	3.721	0.503	0.250	1.011	0.054

* Lower score indicates better perception

*CI* confidence interval

We conducted the multiple (binomial) logistic regression analysis with forward stepwise variable selection to detect predictors of donor satisfaction. Six variables from univariate analyses with *p* < 0.1 were included in the model. Of the six variables, the following three were excluded in forward stepwise variables selection: “increase in self-esteem/self-worth after donation” in Perception of donation results and transplant procedure, donor Self-Rating Depression Scale score, and recipient CSQ-8 score

## Discussion

The CSQ-8 scale has been used extensively within mental health care, primarily medical care, and in a wide range of human service settings [18–20]. For example, mean reported CSQ-8 scores were 25.3 and 22.1 in patients receiving collaborative care versus usual care for depression, respectively, in primary care in the United Kingdom [18]; and mean reported CSQ-8 scores ranged from 26.5 to 27.0 among Filipino women receiving childbirth-related care [19]. These results are in line with the mean CSQ-8 score of 26.9 in the present study.

Decreased perceptions of having received adequate information prior to transplantation was one of the risk factors for lower post-donation satisfaction in this study. Believing that information given preoperatively was inadequate has been reported to be correlated with LKD dissatisfaction [1]. Conversely, in one survey on informed consent among LKDs, donors’ perceptions of understanding the effects of living donation on recipient outcomes was related to the donors’ decision to donate again [25]. Furthermore, recent reviews on psychosocial issues in LKDs have suggested that post-donation feelings of being inadequately informed preoperatively were associated with HRQoL, particularly psychological well-being [15, 26].

Optimism has been reported as having a positive effect on psychological [13] and physical [14] aspects among LKDs, suggesting a positive effect on post-donation satisfaction. A recent large cross-sectional cohort study demonstrated that having lower self-reported optimism was one of the factors that contributed to increased depressive symptoms following kidney donation [13]. Another study demonstrated the positive influence of optimism on wound healing in LKDs [14]. Optimism has been reported to have a positive relationship with increased HRQoL in patients with several illnesses, including those undergoing heart transplantation [27], as well as a positive relationship with increased mental well-being and distress among caregivers of patients with cancer [28].

Recipients’ adverse outcomes may be associated with feelings of waste and guilt [1], depression, as well as conflict in the donor–recipient relationship [29]. It has been reported that LKDs whose paired recipient died within 1 year of transplantation were more likely to state that they would not donate again if repeat donation were possible [2]. Conversely, one study of living liver donors conducted in the United States demonstrated that 100% of donors would donate again and would recommend donation to someone considering organ donation, even though 12% of recipients did not improve after transplantation [30]. In the present study, we did not include recipients with serous negative outcomes, e.g., graft loss or death. Instead, high serum creatinine levels, indicating a poor post-transplant condition in recipients, were found to be related to lower post-donation satisfaction. Poor self-care or nonadherence, which can occur in some recipients, may be associated with dissatisfaction in donors.

Chronic pain has been reported in several studies as an important factor influencing post-donation QOL and satisfaction [9, 31], although laparoscopic surgical procedures have contributed to reducing perioperative pain and discomfort. A recent study has demonstrated that one-quarter of donors who underwent a hand-assisted laparoscopic donor nephrectomy experienced chronic post-donation pain or discomfort, most of which was bothersome [32]. In the present study, however, pain was not associated with donors’ satisfaction.

We must mention an important cultural aspect in this study. Unlike in Western countries, nearly all kidney transplantation in Japan involves living donors; for example, 89.3% of a total 1648 transplanted kidneys in 2016 were from living donors [33]. As we have mentioned, in principle, living organ donors in Japan are limited to family members. Several ethical problems directly related to such family relationships have been identified [16], and the Asian mentality around family has been the subject of debate [17]. However, such relationships with family, including understanding and support from the family, was not associated with post-donation dissatisfaction in the present study.

The strength of this study is that we successfully clarified the risk factors for low post-donation satisfaction using a valid instrument. In addition, this study may contribute to the understanding of decision-making and satisfaction among LKDs in the context of Asian family relationships.

The study does, however, have certain limitations. First, we primarily recruited recipients who were followed up in a post-transplant outpatient service, and we asked recipients if their donors would participate in this study. Therefore, recipients who left the outpatient service owing to worsened outcome (e.g., returning to hemodialysis after graft loss, or death) were not included in this study; thus, donors paired with such recipients were not included. Although we recommended that all donors undergo regular follow-up at our transplant center, this was not necessarily adhered to by all donors because the patient may have been too old to visit the clinic, they lived far away, or they were followed by their primary physician. Second, if recipients did not want their paired donor to participate in this study, these donors were excluded from this study. Third, like all such surveys, ours was subject to self-selection bias owing to the donors themselves. Indeed, it is possible that issues beyond those discussed here exist among such donors. Fourth, because this study was carried out at a single center located in metropolitan Tokyo, our findings may not be applicable to people living in other areas of Japan; for instance, nuclear families are more frequent in urban than in rural areas. Fifth, because study participants were limited to Japanese people, our findings may not be applicable to other ethnic groups. Sixth, other possible mediating factors associated with dissatisfaction were not tested, e.g., psychological traits other than depression or optimism. Finally, the number of respondents was relatively low for the identification of risk factors for low satisfaction among LKDs.

Finally, we used the CSQ-8, a standardized scale for global client/patient satisfaction with health services and clinical care, to evaluate post-donation satisfaction levels in the present study. However, donor satisfaction has been reported to be multifaceted. For example, as mentioned, Menjivar et al. [9] suggested that donor satisfaction seems better characterized according to the following three dimensions: unmet donor expectations about donation; interference of donation with daily activities, and pain and discomfort. Therefore, the methodology in donor satisfaction research should be further considered based on the results of such qualitative studies.

To overcome several of these limitations, prospective, multi-facet studies focusing on post-donation satisfaction among LKDs that use standardized tools, such as those used in this study, may be required in the future.

## Conclusions

In this cross-sectional study of post-donation satisfaction among LKDs using a validated instrument, the CSQ-8, we identified three risk factors: (1) a perception of receiving inadequate prior information, (2) donor pessimism, and (3) poor post-transplant physical condition of the recipient, as indicated by high serum creatinine levels. Our findings suggest that careful pre-donation education and more detailed informed consent may be needed, especially among LKDs with low constitutional optimism.

## Supplementary information

**Additional file 1.** Donors’ perception of donation results and transplant procedure. A 13-item scale developed from a qualitative study of potential LKDs on the factors influencing decision-making when considering donation.

## Data Availability

The datasets used and/or analyzed during the current study are available from the corresponding author on reasonable request.

## Abbreviations

CI: Confidence interval
CSQ-8: Client Satisfaction Questionnaire-8
HRQoL: Health-related quality of life
LKD: Living kidney donor
LOT: Life Orientation Test
OR: Odds ratio
SD: Standard deviation
SDS: Zung Self-Rating Depression Scale
SF-36: Short Form-36 Health Survey
