# Supplementary material for: Post-donation satisfaction in kidney transplantation: a survey of living donors in Japan
Source: BMC Health Serv Res. 2019 Oct 26;19:755. doi: 10.1186/s12913-019-4556-5 (PMC6815382; doi:10.1186/s12913-019-4556-5)
Supplement: Supplementary file 1 — Additional file 1. Donors’ perception of donation results and transplant procedure. A 13-item scale developed from a qualitative study of potential LKDs on the factors influencing decision-making when considering donation. [file 12913_2019_4556_MOESM1_ESM.docx]

**Additional file 1**

**Donors’ perception of donation results and transplant procedure**

**Factor 1**. Good relationship with and support from family members

*My family was supportive of my donation.*

*My family respected my feelings and thoughts regarding donation.*

*I am on good terms with family members (other than the recipient).*

**Factor 2**. Adequate information prior to transplant

*I was given enough information by the hospital.*

*I received enough information regarding donation before the operation.*

*I trust the hospital and the doctor who operated on me.*

**Factor 3**. Recipient’s recovery

*The recipient has recovered their health.*

*The recipient can get on with their life and job.*

**Factor 4**. Recipient’s gratitude toward the donor

*I think the recipient is happy with my donation.*

*I think the recipient is grateful to me for the donation.*

**Factor 5**. Increased self-esteem/self-worth after donation

*It was good that I donated and not someone else.*

*I would rather have had someone else donate.*

*Donation is compatible with my values (ideas about life or religious beliefs).*
